# Quantitative 7-Tesla Imaging of Cortical Myelin Changes in Early Multiple Sclerosis

**DOI:** 10.3389/fneur.2021.714820

**Published:** 2021-09-03

**Authors:** Valeria Barletta, Elena Herranz, Constantina A. Treaba, Ambica Mehndiratta, Russell Ouellette, Gabriel Mangeat, Tobias Granberg, Jacob A. Sloane, Eric C. Klawiter, Julien Cohen-Adad, Caterina Mainero

**Affiliations:** ^1^Athinoula A. Martinos Center for Biomedical Imaging, Department of Radiology, Massachusetts General Hospital, Boston, MA, United States; ^2^Harvard Medical School, Harvard University, Boston, MA, United States; ^3^Department of Clinical Neuroscience, Karolinska Institutet, Stockholm, Sweden; ^4^Department of Neuroradiology, Karolinska University Hospital, Stockholm, Sweden; ^5^NeuroPoly Lab, Institute of Biomedical Engineering, Polytechnique Montreal, Montreal, QC, Canada; ^6^Department of Neurology, Beth Israel Deaconess Medical Center, Boston, MA, United States

**Keywords:** quantitative MRI, cortical demyelination, cortical remyelination, NODDI, early multiple sclerosis

## Abstract

Cortical demyelination occurs early in multiple sclerosis (MS) and relates to disease outcome. The brain cortex has endogenous propensity for remyelination as proven from histopathology study. In this study, we aimed at characterizing cortical microstructural abnormalities related to myelin content by applying a novel quantitative MRI technique in early MS. A combined myelin estimation (CME) cortical map was obtained from quantitative 7-Tesla (7T) T${}_{2}^{*}$ and T_1_ acquisitions in 25 patients with early MS and 19 healthy volunteers. Cortical lesions in MS patients were classified based on their myelin content by comparison with CME values in healthy controls as demyelinated, partially demyelinated, or non-demyelinated. At follow-up, we registered changes in cortical lesions as increased, decreased, or stable CME. Vertex-wise analysis compared cortical CME in the normal-appearing cortex in 25 MS patients vs. 19 healthy controls at baseline and investigated longitudinal changes at 1 year in 10 MS patients. Measurements from the neurite orientation dispersion and density imaging (NODDI) diffusion model were obtained to account for cortical neurite/dendrite loss at baseline and follow-up. Finally, CME maps were correlated with clinical metrics. CME was overall low in cortical lesions (*p* = 0.03) and several normal-appearing cortical areas (*p* < 0.05) in the absence of NODDI abnormalities. Individual cortical lesion analysis revealed, however, heterogeneous CME patterns from extensive to partial or absent demyelination. At follow-up, CME overall decreased in cortical lesions and non-lesioned cortex, with few areas showing an increase (*p* < 0.05). Cortical CME maps correlated with processing speed in several areas across the cortex. In conclusion, CME allows detection of cortical microstructural changes related to coexisting demyelination and remyelination since the early phases of MS, and shows to be more sensitive than NODDI and relates to cognitive performance.

## Introduction

Demyelinating cortical lesions are a pathologic hallmark of multiple sclerosis (MS), which can develop from the earliest disease stages and relate to disease progression (1–6).

Based on the presence of myelin degradation products inside macrophages and microglia, cortical lesions can be distinguished as actively demyelinating and postdemyelinating (4, 7). Ongoing demyelination can coexist within the same lesion with massive remyelination (8); the efficiency of the latter seemingly changes according to disease type and anatomical location (9, 10).

*Ex vivo* MS examinations show that remyelination is more effective in cortical than in white matter (WM) lesions, possibly due to higher availability of oligodendrocytes and their precursors (10, 11). Cortical remyelination occurs with a heterogeneous pattern, ranging from extensive (~18% of lesions) to mostly peripheral in the majority of lesions (10). Pathology studies, however, are skewed toward late MS cases, often with associated comorbidities. Knowledge on remyelinating phenomena in the early disease is, thus, very limited, such as the actual clinical impact of cortical demyelination and remyelination in MS patients.

The combined myelin estimation (CME) model extracts the shared information related to myelin content from different quantitative and semiquantitative MRI contrasts, limiting confounding factors such as iron, cortical thickness, and B_0_ inhomogeneity (12). CME maps obtained from 7-Tesla (7T) quantitative T${}_{2}^{*}$ and 3-Tesla (3T) magnetization transfer ratio in healthy individuals have previously shown spatial distribution similar to that from histological work stained for myelin (13). More recently, CME obtained from quantitative 7T ultra high-resolution T${}_{2}^{*}$ and T_1_ maps has been proven to have excellent scan-rescan reproducibility and increased sensitivity to cortical MS pathology compared to individual maps (14).

We hypothesized that cortical microstructural alterations, likely related to myelin content abnormalities, could be measured *in vivo* using CME from 7T quantitative T${}_{2}^{*}$ and T_1_ mapping in a cohort of 25 early MS cases relative to 19 age-matched healthy volunteers in both lesioned and normal-appearing cortex and would evolve dynamically, suggesting cortical demyelination and remyelination phenomena. As myelin content could be influenced by additional pathological factors including neurite/dendrite loss, measurements from the neurite orientation dispersion and density imaging (NODDI) diffusion model (15–17) were obtained along CME in cortical lesions and whole cortex. Secondarily, the relevance of CME abnormalities on clinical and radiological estimates of disease burden was investigated. Finally, to further validate the CME technique, we assessed the relation between CME values in the healthy subjects and *ex vivo* myelin density optical measurements (18) across several Brodmann areas.

## Materials and Methods

### Subjects and Study Procedures

The Institutional Review Board approved this prospective study, and all subjects gave written informed consent to participate. The work described here has been carried out in accordance with the Declaration of Helsinki.

Twenty-five early MS patients and 19 age-matched healthy volunteers were enrolled between Match 2014 and March 2019. General inclusion criteria were the following: age 18–60, no significant medical history (other than MS for patients), and no MRI contraindications. Inclusion criteria for MS were relapsing–remitting MS diagnosis according to the McDonald criteria (19), disease duration ≤ 5 years, stable disease-modifying treatment or no treatment and no relapses in the 3 months prior to enrolment, and no corticosteroid use for 1 month prior to enrolment.

At baseline and follow-up, within a week from MRI, patients underwent neurological examination with assessment of the Expanded Disability Status Scale (EDSS) score and Symbol Digit Modalities Test (SDMT). One patient could not perform SDMT because of severe visual impairment. SDMT raw scores were converted to Z-scores (SDMT-z) after correcting for age and education (20).

**Figure 1:**
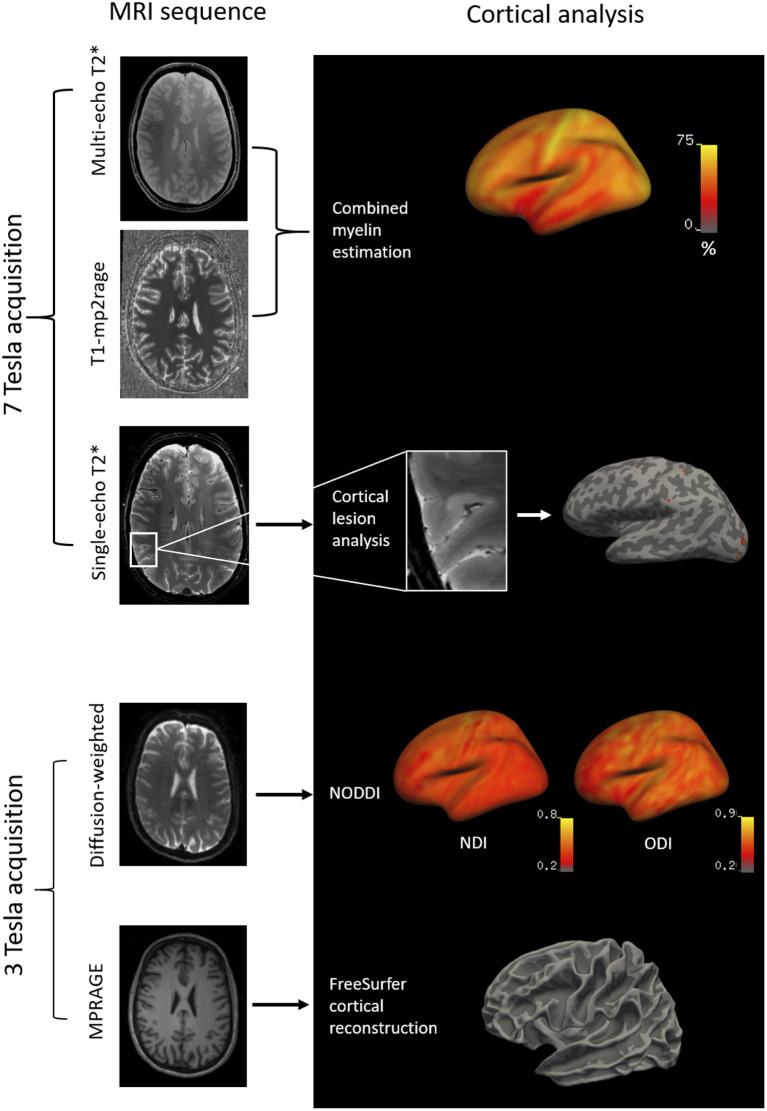
Methodology of the imaging analysis: 7T multi-echo T_1_-MP2RAGE and T${}_{2}^{*}$ sequences were combined by independent component analysis to obtain cortical CME maps. Cortical lesions were segmented on 7T single-echo T${}_{2}^{*}$ sequences and projected to the surface of the subject. Diffusion-weighted images at 3T (b-values=1,000 and 5,000 s/mm^2^; diffusion directions=64 and 128) were acquired to obtain cortical NODDI maps. T1-weighted MPRAGE images at 3T were acquired for FreeSurfer cortical reconstructions. MP2RAGE, dual magnetization-prepared rapid gradient echo; MPRAGE, magnetization prepared rapid acquisition gradient echo.

All subjects were scanned on a 7T MRI whole-body scanner (MAGNETOM, Siemens Healthcare, Erlangen, Germany) equipped with a 32-channel receive head coil, and on a 3T whole-body MRI scanner (MAGNETOM Skyra CONNECTOM, Siemens Healthcare, Erlangen, Germany) using a 64-channel head coil within 1 week from the 7T scan. Ten MS patients (mean age, 36 ± 7.6 years; females: 8) were rescanned with the same protocols after 1 year (mean follow-up time, 1.0 ± 0.2 years). Detailed MRI protocol is reported in Table 1. Figure 1 summarizes the imaging procedures.

**Table 1:** MRI sequence protocol.

**Sequence**	**Field strength**	**Parameters**
Single-echo two-dimensional (2D) fast low-angle shot T${}_{2}^{*}$-weighted spoiled gradient echo	7T	Voxel size = 0.33 × 0.33 × 1 mm; repetition time = 1,700 ms; echo time = 21.8 ms; acquisition time = ~7.5 min per slab (2 slabs)
Multi-echo 2D T${}_{2}^{*}$ gradient echo	7T	Voxel size = 0.5 mm isotropic; repetition time = 3,680 ms; echo time = 3.12+3.32*[1.6] ms; acquisition time = ~10 min per slab (2 slabs); number of echoes = 6
3D dual magnetization-prepared rapid gradient echo (MP2RAGE)	7T	Voxel size = 0.75 mm isotropic; repetition time = 5,000 ms; echo time = 2.93 ms; inversion time = 900–3,200 ms; acquisition time = 10 min; flip angles = 4° and 5°
3D T_1_-weighted multi-echo magnetization prepared rapid acquisition gradient echo (MPRAGE)	3T	Voxel size = 1 mm isotropic; repetition time = 2,530 ms; echo time = 1.15, 3.03, 4.89, 6.75; acquisition time = 6 min TI = 1,100 ms
2D echo-planar multishell diffusion weighted	3T	Voxel size = 1.5 mm isotropic; b-values = 1,000 and 5,000 s/mm^2^; diffusion directions = 64 and 128; acquisition time = ~12 min/22 min

### Cortical Surface Reconstruction

Cortical surface reconstruction and cortical thickness measurements were performed on the 3T MPRAGE using FreeSurfer v5.3.0 (http://freesurfer.net) after correction for gradient non-linearity-induced distortions. Topological surface reconstruction defects caused by lesions were corrected with lesion in-painting on FreeSurfer.

### CME and Diffusion Modeling

Multilinear regressions were performed using predictors of myelin content (T_1_ and T${}_{2}^{*}$) and a confounding covariate (cortical thickness, which can introduce variable amount of partial volume effect). Secondly, a spatial independent component analysis was used to extract the myelin-specific signal shared by the T_1_ and T${}_{2}^{*}$ maps to obtain the CME % map (14). A detailed mathematical description of the model has been previously published (13).

Diffusion-weighted images were preprocessed as previously detailed (17), and then analyzed using the NODDI toolbox (v. 0.9, University College London, London, UK; http://mig.cs.ucl.ac.uk/mig/mig/index.php/?n=Tutorial.NODDImatlab/) (15) to obtain maps of neurite density index (NDI) and orientation dispersion index (ODI), a measure of orientational coherence or structural integrity. Maps of CME and NODDI were projected to the cortical surface.

Maps of CME were sampled at 25, 50, and 75% depth. Maps of NDI and ODI were sampled at 50% depth to minimize partial volume effects (17). Individual surfaces of CME and NODDI metrics were registered to a common template surface in FreeSurfer and smoothed with a full width at half-maximum kernel of 5 and 10 mm, respectively.

### Lesion Quantification and Analysis

Intracortical, leukocortical, and WM lesions were manually segmented on Slicer (version 4.4.0, 2014; http://www.slicer.org) on T${}_{2}^{*}$-weighted single echo images and checked on the corresponding T_1_ maps from 7T MP2RAGE acquisitions by active collaboration of one radiologist and one neurologist (CT and CM). New lesions were identified on a lesion-by-lesion basis through agreement. Lesions were identified as focal hyperintensities that extended for at least three voxels and across two consecutive slices (21). Lesion volumes were quantified by FSL using fslstats from the FMRIB Software Library, v. 5.0 (http://fsl.fmrib.ox.ac.uk/fsl). Masks of normal-appearing cortex were obtained by subtracting the lesion masks from the whole cortex by using fslmaths on FSL.

Values for CME and NODDI were extracted at 50% cortical depth in cortical lesion masks and normal-appearing cortex. Since the focus of the study was the cortex, CME and NODDI for leukocortical lesions were measured only in the intracortical portion of the mask.

At baseline, myelination status for each cortical lesion was defined based on the percentage of voxels that had CME values ≤ 2 SD of the mean CME value in the corresponding cortical area in the control group using the Desikan-Killiany atlas in FreeSurfer. This was done to account for differences in myeloarchitecture across the cortex. Based on the percentage of voxels with reduced CME within an individual lesion, cortical lesions were then classified as demyelinated (≥50%), partially demyelinated (5–49%), or non-demyelinated (<5%). At follow-up, we registered significant changes in CME within each individual lesion as a variation of at least 1 SD from the basal value (22) (increased, decreased, or stable CME).

### Statistical Analysis

Demographic data were compared between patients and controls by Wilcoxon test. Multilinear regression with age and gender as nuisance factors was used to compare cortical thickness in patients vs. healthy volunteers. CME measures in the healthy cohort were compared by Pearson's correlation to optic myelin measurements previously performed histologically (13, 18).

Matched paired *t*-test was used to assess (i) CME and NODDI in cortical lesions vs. normal-appearing homologous contralateral gray matter, (ii) changes in cortical lesion CME and NODDI at follow-up, (iii) changes in cortical lesion and WM lesion volume at follow-up, and (iv) changes in EDSS and SDMT-z at follow-up. A multilinear regression, including age as a nuisance factor, was used to compare CME in cortical lesions vs. cortical gray matter in healthy controls.

Nominal logistic regression was used to correlate CME status at baseline (demyelinated, partially demyelinated, or non-demyelinated) to NODDI continuous values and to correlate CME status at follow-up (increased, decreased, or stable) with lesion type, while multilinear regression was applied to compare NODDI at baseline and follow-up in individual lesions divided based on CME status at follow-up.

To correlate changes in CME with changes in clinical metrics at follow-up, the variation in CME, EDSS, and SDMT-z was assessed (follow-up-baseline value) and related through Pearson's correlation.

Spearman's correlation was used to relate cortical CME to WML and cortical lesion load.

A vertex-wise general linear model (GLM) on FreeSurfer was used to (i) assess differences in cortical CME and NODDI between patients and controls, (ii) assess longitudinal CME changes in patients, (iii) correlate cortical CME with EDSS and SDMT-z, and (iv) correlate cortical thickness with EDSS and SDMT-z. In all GLM analyses, a cluster-wise correction for multiple comparisons using Monte Carlo simulation with 10,000 iterations was applied. Significant clusters were localized using the Desikan-Killiany atlas in FreeSurfer. Since the cortical thickness map was used to construct the CME cortical map, the vertex-wise between the two metrics was not investigated, but cortical thickness was included in all CME GLM analyses as a vertex-wise covariate of no interest.

Statistical analysis was performed on JMP pro v13. A *p* < 0.05 was considered significant.

## Results

### Demographic, Clinical, and MRI Results

The demographic, clinical, and MRI characteristics of the study subjects are summarized in Table 2.

**Table 2:** Demographic, clinical and radiological characteristics of the study subjects.

**Characteristic**	**Healthy controls**	**MS patients**	***p***
	***n* = 19**	***n* = 25**	
Age, mean ± SD	35.8 ± 10.2	38.2 ± 8.9	0.3^a^
Females, number	10	21	0.04^b^
Disease duration in years, median (range)	–	1.9 (0.6-4.7)	–
Age at disease onset, mean ± SD	–	35 ± 8.1	-
EDSS, median (range)	–	2 (0–4)	-
SDMT z-score, median (range)	–	0.75 (−1.92, 3.3)	–
Disease modifying treatment *n*	–	22	–
- Dimethyl fumarate *n*	–	10	–
- Glatiramer acetate *n*	–	5	–
- Interferon beta-1a *n*	–	4	–
- Natalizumab *n*	–	2	–
- Ocrelizumab *n*	–	1	–
Cortical thickness in mm, mean ± SD	2.43 ± 0.1	2.40 ± 0.1	0.89^c^
Total cortical lesion count, median (range)	–	2(1–2)	–
Total cortical lesion volume mm^3^, mean±SD	–	245 ± 245	–
Intracortical lesion volume mm^3^, mean±SD	–	162 ± 161	–
Leukocortical lesion volume mm^3^, mean±SD	–	164 ± 180	–
WM lesion volume mm^3^, mean±SD	–	1,357 ± 2160	–

*SD, standard deviation.*

a *By Wilcoxon test.*

b *By Fisher's exact test.*

c *By multilinear regression correcting for age and gender*.

**Figure 2:**
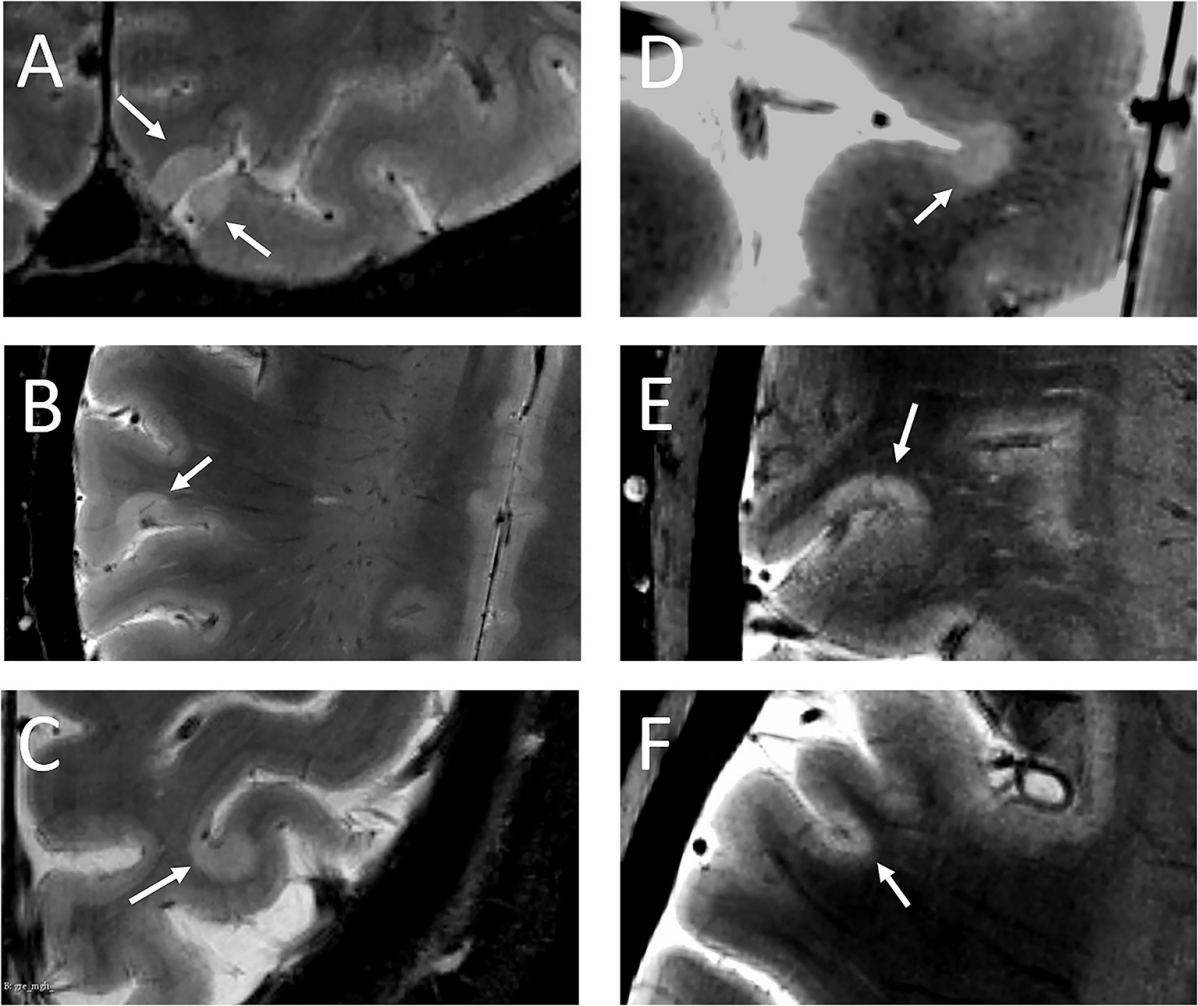
White arrows indicate leukocortical **(A–C)** and intracortical **(D–F)** lesions detected in the MS cohort.

No lesions were detected in the healthy group. At baseline, 103 cortical lesions were detected in 20 of 25 (80%) MS patients: 62 (60%) intracortical, 41 (40%) leukocortical (Table 2, Figures 2A–F).

At 1-year follow-up, 25 new cortical lesions (23 intracortical and 2 leukocortical) developed in 4 of 10 patients. Overall, both cortical and WM lesion volumes tended to be higher at follow-up, although not significantly. Cortical thickness did not change significantly (Table 3).

**Table 3:** MRI characteristics of the MS patients rescanned after 1 year.

**MRI characteristic**	**Baseline**	**Follow-up**	***p***
Cortical thickness mm, mean ± SD	2.40 ± 0.08	2.41 ± 0.07	0.67^a^
Total cortical lesion count, median (range)	5 (1–22)	5.5 (1–33)	–
WM lesion volume mm^3^, mean ± SD	1,350 ± 2,162	1,472 ± 2,025	0.3^a^
Total cortical lesion volume mm^3^, mean ± SD	253 ± 242	369 ± 566	0.4^a^

*SD, standard deviation.*

a *By matched pairs t-test*.

Diffusion NODDI images were acquired at baseline in 21 MS patients (18 of which showed cortical lesions) and 17 healthy controls, and in 9 of 10 patients rescanned at 1-year follow-up.

### CME and NODDI Metrics Along the Normal-Appearing Cortex

**Figure 3:**
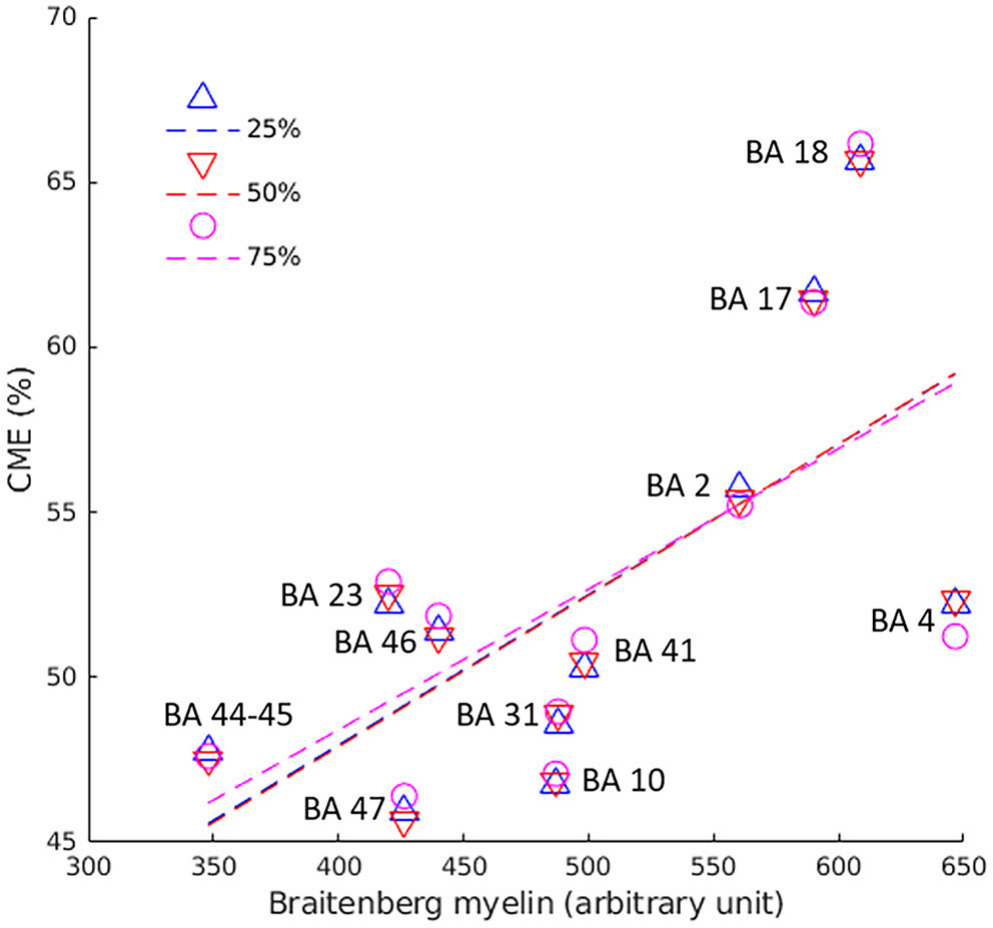
Pearson's correlation between CME values in the cortex and myelin optic measurements across 12 BA of healthy controls. Pearson's coefficient was 0.6 (*p* = 0.036) at 25% (blue triangles/line), 0.7 (*p* = 0.022) at 50% (red triangles/line), and 0.7 (*p* = 0.024) at 75% (pink circles/line) of cortical depth from the pial surface. BA, Broadmann area.

In healthy subjects, we found a positive correlation between CME at each cortical depth and myelin density optical measurements previously obtained in the corresponding cortical regions (13, 18) of a healthy adult human brain (by Pearson's correlation; Figure 3).

**Figure 4:**
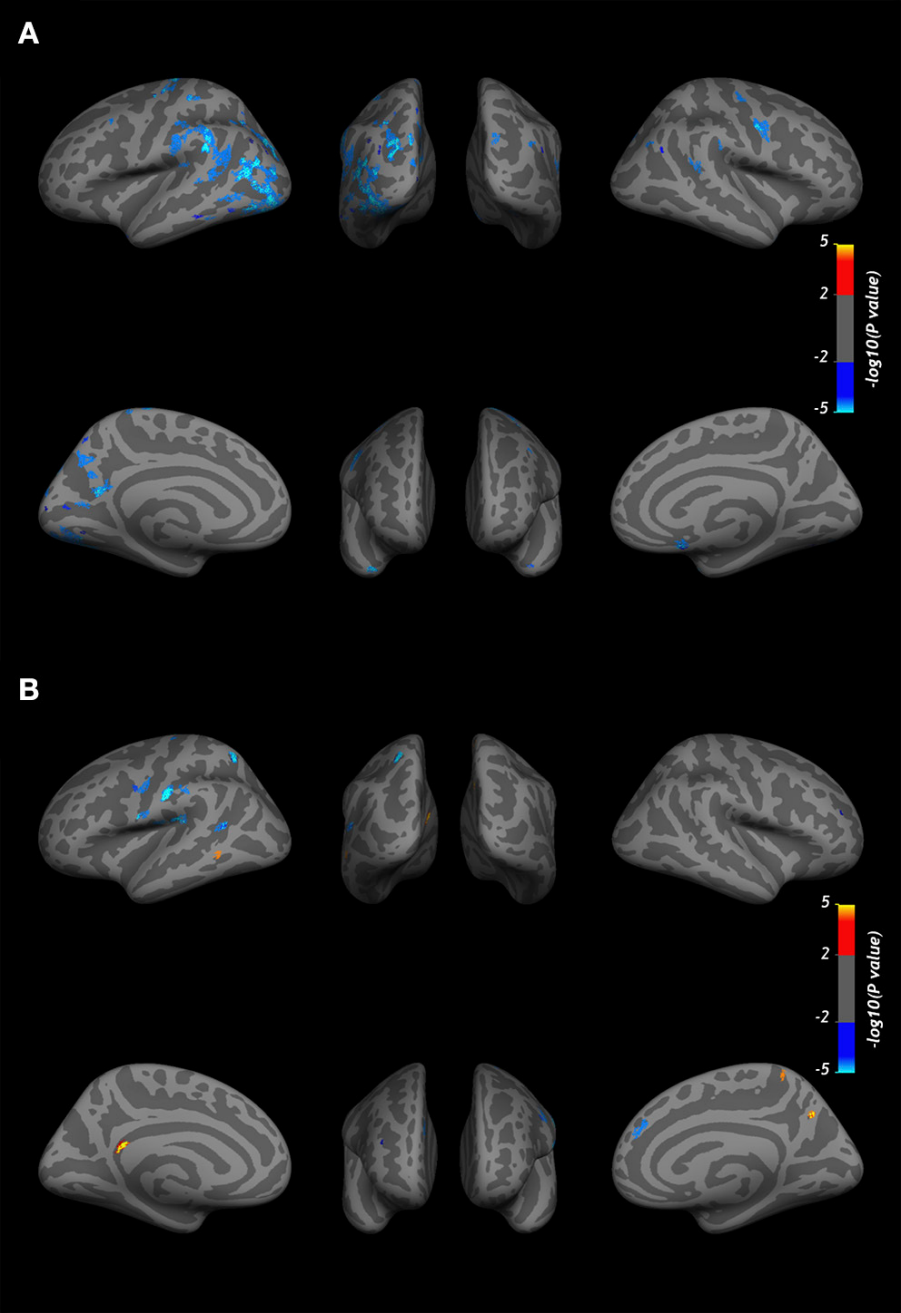
Overlay of the GLM significance map (*p* < 0.05 corrected for multiple comparisons). **(A)** Clusters of significantly reduced CME in the normal-appearing cortex of MS patients vs. healthy controls. **(B)** Variation in CME values in the normal-appearing cortex of 10 MS patients rescanned at 1-year follow-up. Blue clusters indicate areas of CME reduction, and yellow clusters indicate CME increase. Both analyses were performed on normal-appearing cortex at three cortical depths (25, 50, and 75%) from the pial surface, overlapped in the figure. Color bars show -log10(p-value).

At baseline, the GLM analysis revealed several clusters of significantly reduced CME in MS patients compared to the control group in the normal-appearing cortex at all cortical depths (Figure 4A). Table 4 summarizes the localization of the clusters in which CME values were significantly different in MS patients vs. controls. No differences in NDI or ODI were seen at GLM analysis between patients and controls along the whole cortex.

**Table 4:** Localization of clusters indicating lower cortical CME in MS patients vs. healthy subjects at three cortical depths.

**Left hemisphere**	
25% from pial surface	Fusiform, precentral
50% from pial surface	Lateral occipital (387 mm^2^), inferior parietal (282 mm^2^), superior parietal (179 mm^2^), supramarginal (119 mm^2^), precentral, precuneus, fusiform, postcentral, temporal pole, lingual gyrus
75% from pial surface	Lingual gyrus (1882 mm^2^), supramarginal (962 mm^2^), superior parietal (390 mm^2^), inferior parietal (337 mm^2^), precentral (309 mm^2^), precuneus (281 mm^2^), peri-calcarine (186 mm^2^), inferior temporal (157 mm^2^), banks of the superior temporal sulcus (124 mm^2^), postcentral, cuneus, paracentral, caudal middle frontal, middle temporal, superior frontal
**Right hemisphere**	
25% from pial surface	Temporal pole, precentral
50% from pial surface	Temporal pole, medial orbito-frontal, fusiform
75% from pial surface	Precentral (307 mm^2^), superior parietal, banks of the superior temporal sulcus, inferior parietal, caudal anterior cingulate, fusiform, supramarginal, temporal pole

*Total area is indicated in brackets for clusters with area ≥100 mm^2^*.

In 10 patients rescanned at the 1-year follow-up, CME % was overall reduced in normal-appearing cortex (50.0 ± 2 vs. 49.7 ± 2, *p* = 0.02 by matched pairs *t*-test). The vertex-wise comparison showed, however, fewer clusters with increase in CME at follow-up (Figure 4B).

### CME and NODDI Metrics in Cortical Lesions

Overall, cortical lesions showed lower mean CME values relative to the contralateral normal-appearing cortex (*p* = 0.04 by *t*-test) and to cortex of healthy subjects (*p* = 0.03 by multilinear regression), while no differences were detected for either NDI or ODI in the same regions (Table 5).

**Table 5:** Results from the CME and neurite orientation dispersion and density index modeling in the cortex of early stage MS subjects and healthy controls.

**Metric**	**MS cortical lesions**	**MS contralateral normal appearing cortex**	***p***	**MS whole cortex**	**Healthy controls whole cortex**	***p***
CME % mean ± SD	47.1 ± 6.4	50.0 ± 8.6	0.04^a^	50.2 ± 1.8	50.6 ± 1.8	0.4^b^
NDI mean ± SD	0.42 ± 0.03	0.42 ± 0.03	0.4^a^	0.43 ± 0.01	0.43 ± 0.02	0.8^b^
ODI mean ± SD	0.55 ± 0.04	0.56 ± 0.05	0.6^a^	0.56 ± 0.01	0.56 ± 0.01	1.0^b^

a *By matched pairs t-test.*

b *By multilinear regression (adjusted for age)*.

Individual cortical lesion analysis showed that 62 of 103 (60%) of them were demyelinated, either extensively (33/103, 32%) or partially (29/103, 28%). The percentage of demyelination did not relate to lesion type (*p* = 0.4 by Wilcoxon test for intracortical vs. leukocortical). Forty-one out of 103 lesions showed <5% demyelinated voxels.

Values of NDI and ODI were extracted individually for 97 cortical lesions (18 MS subjects) at baseline. No difference was seen in either NDI or ODI in MS cortical lesions relative to the contralateral normal-appearing cortex in patients (respectively, *p* = 0.4 and 0.6, by *t*-test) and to the cortex of healthy controls (*p* = 0.3 and 0.2 by multilinear regression) (Table 5). Values of NDI and ODI were also grouped based on cortical lesion myelination status as measured by CME and did not show any difference between demyelinated, partially demyelinated, or non-demyelinated cortical plaques (for NDI, *p* = 0.6; for ODI, *p* = 0.7, by nominal logistic regression; subject ID was an adjusting factor).

At follow-up, cortical lesions showed an overall reduction in CME % (48.8 ± 7.2 vs. 46.5 ± 6.1, *p* = 0.03 by *t*-test), suggesting further global demyelination.

Individual CME lesion analysis revealed that out of the total 63 lesions reexamined at follow-up, 26 (41%) showed reduction in their basal CME values >1 SD, 27 (43%) showed no significant changes, while 10 (16%) registered an increase ≥1 SD of CME basal values. Interestingly, two of the lesions with increased CME were fully demyelinated at baseline but reached mean CME values in the range of normality at follow-up, suggesting effective remyelination.

Lesion type (intracortical or leukocortical) was not a predictor of CME changes at follow up (*p* = 0.59 by nominal logistic regression).

No significant changes were seen in NODDI metrics in cortical lesions (by paired *t*-test) at follow-up. However, when individual cortical lesions were divided based on CME changes at follow-up (increased, stable, or decreased), a significant increase was seen in ODI in lesions with increased CME (ODI mean ± SD, 0.49 ± 0.08 at baseline vs. 0.57 ± 0.02 at follow-up, *p* = 0.009 by multilinear regression; subject ID was included as a confounding factor).

### Correlations With Clinical and MRI Disease Burden

**Figure 5:**
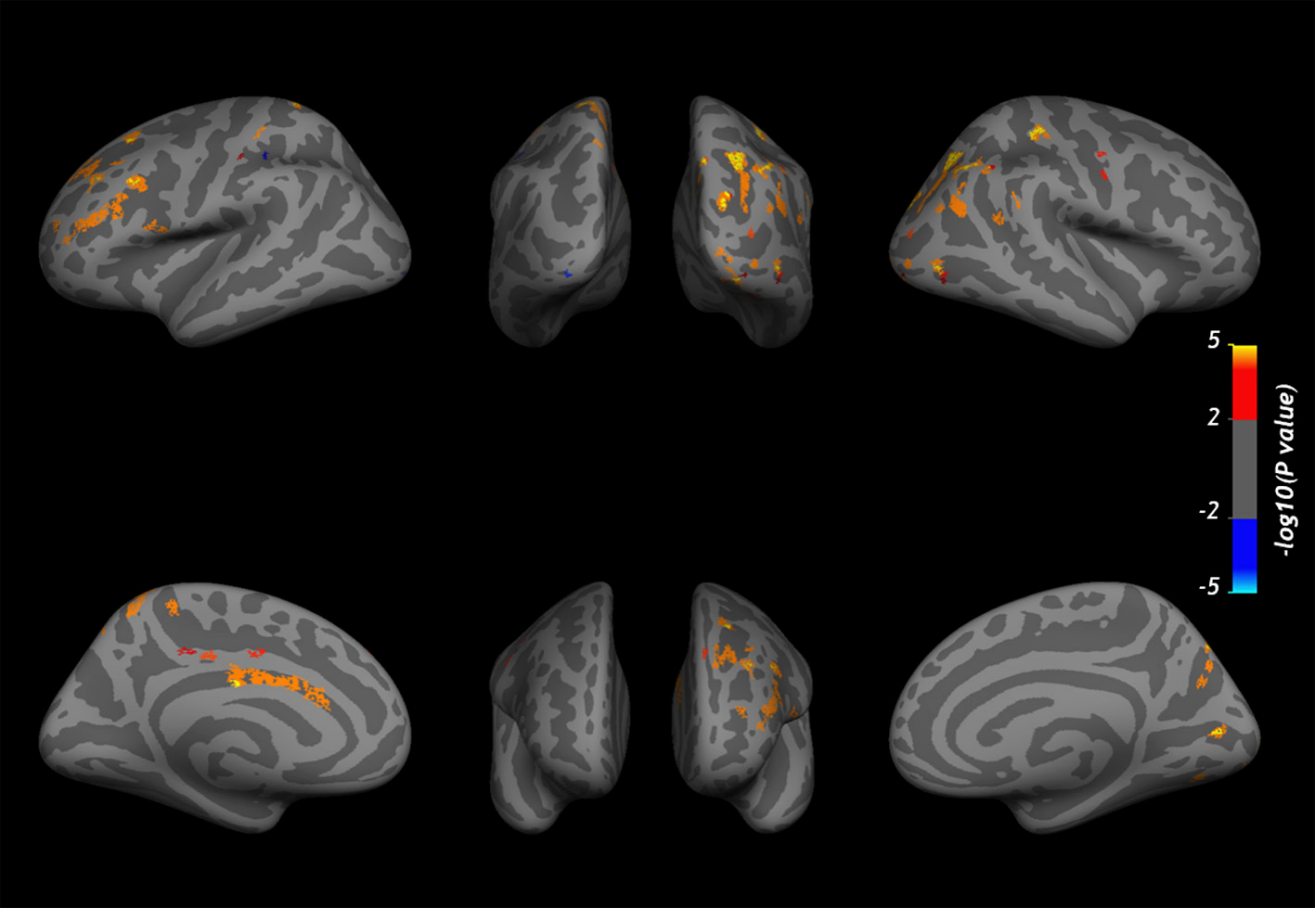
Overlay of the GLM significance map (*p* < 0.05 corrected for multiple comparisons) showing vertex-wise positive correlations between CME and SDMT-z at three cortical depths in MS patients. Color bar shows -log10 (p-value). Cortical thickness and WM lesion load were included in the analysis as adjusting factors.

Vertex-wise positive correlations were found between cortical CME and SDMT-z (Figure 5), while no clusters were found for EDSS. For SDMT-z, positive correlations with myelin content were found mostly at the mid-cortical level in both hemispheres. In the right hemisphere, most clusters were found in the superior and inferior parietal, lateral occipital, and postcentral cortex. In the left hemisphere, clusters were found in the rostral middle frontal, superior frontal, superior parietal, and posterior cingulate cortex. Table 6 indicates the localization of clusters of correlation between CME and SDMT-z at three cortical depths.

**Table 6:** Localization of clusters indicating positive correlation between cortical CME and SDMT z-scores.

**Left hemisphere**	
25% from pial surface	Caudal middle frontal, pars opercularis, rostral middle frontal, postcentral, superior parietal, superior frontal, supramarginal, posterior cingulate
50% from pial surface	Rostral middle frontal (384 mm^2^), superior frontal (285 mm^2^), superior parietal (281 mm^2^), posterior cingulate (236 mm^2^), caudal middle frontal (135 mm^2^), caudal anterior cingulate, para-central, pars opercularis, lateral occipital, isthmus cingulate
75% from pial surface	None
**Right hemisphere**	
25% from pial surface	Superior parietal, postcentral, inferior parietal, precentral, superior frontal, peri-calcarine
50% from pial surface	Superior parietal (446 mm^2^), inferior parietal (365 mm^2^), lateral occipital (364 mm^2^), postcentral (120 mm^2^), precuneus, peri-calcarine, lingual, fusiform, supramarginal, precentral
75% from pial surface	Inferior parietal (125 mm^2^), lateral occipital (100 mm^2^), superior parietal, superior temporal

*Total area is indicated in brackets for clusters with area ≥100 mm^2^*.

Neither EDSS nor SDMT-z registered significant changes at follow-up (for EDSS (mean ± SD): at baseline 1.7 ± 0.8, at follow-up 1.7 ± 0.7, *p* = 0.8; for SDMT-z (mean ± SD): at baseline 0.9 ± 1.1, at follow-up 0.7 ± 1.3, *p* = 0.5 by matched pair *t*-test). Percentage changes in lesion CME did not correlate with changes in EDSS or SDMT-z (respectively, *p* = 0.3 and 0.9, by Pearson's).

No correlations were found between cortical thickness and either EDSS or SDMT-z. There was no correlation between CME and WM or cortical lesion volume.

## Discussion

In this study, we applied CME from quantitative 7T acquisitions to assess microstructural changes likely related to myelin content in cortical lesions and normal-appearing cortex of early MS cases. To account for the possible influence of neural loss on CME, NODDI measurements were also obtained. In patients, mean CME was overall abnormally decreased in cortical lesions and several regions of the cortical mantle, without significant changes in diffusion metrics or cortical thinning, suggesting early cortical demyelination in the absence of decreased neuroaxonal density and overt cortical atrophy. Individual cortical lesion analysis, however, revealed heterogeneous CME patterns, possibly reflecting underlying concomitant phenomena of demyelination, incomplete demyelination, or remyelination.

Cortical CME in the control group correlated across several Brodmann areas with myelin optical measurements obtained from histology (18), providing indirect evidence that the metric is related to cortical myelin content. Compared to previous CME data obtained by combining 7T T${}_{2}^{*}$ with 3T magnetization transfer ratio (13), the current approach enables acquisition of higher-resolution images, which is more effective for laminar cortical pathology analysis.

Cortical lesions were present in 80% of MS subjects, confirming their high detection in early MS *in vivo* by 7T MRI (17, 23, 24).

Overall CME was reduced both in cortical lesions and normal-appearing cortex, suggesting the presence of widespread microstructural abnormalities related to demyelination. When we performed an individual cortical lesion analysis, we found that 60% of cortical lesions had some degree of demyelination, while the remaining had reduced CME in <5% of the total voxels. This pattern is supported by previous combined histopathological/MRI analyses of autoptic cortical MS tissue, which showed that some of the cortical hyperintensities detected by 7T T${}_{2}^{*}$ sequences (7/83, 8%) represented incompletely demyelinated or remyelinating areas (24).

Despite an overall decrease in lesion CME at the 1-year follow-up, significant increase in few cortical lesions and some areas in the normal-appearing cortex was detected, suggesting the presence of both demyelination and remyelination processes, as observed in histopathology studies (9–11, 25).

The results from the NODDI analysis showed that neither NDI nor ODI was altered in early MS patients, while CME showed significant differences both in cortical lesions and in normal-appearing cortex, proving to be very sensitive in disclosing early microstructural damage.

The NDI relates to neuroaxonal density and has been found to be reduced in the MS WM and cortex, especially in the progressive disease (16, 17, 26). In *ex vivo* studies, NDI has also been found to correlate with myelin content (16, 27). Our results suggest that, at least in our cohort, cortical demyelination is not accompanied by neural loss in the early phase of the disease and that CME seems sensitive in detecting early cortical myelin changes. Additionally, relative to NODDI, the CME approach from 7T MRI enables acquisition of higher-resolution images, which may be more effective for laminar cortical pathology analysis.

The higher ODI found in cortical lesions with increased CME at follow-up could indicate higher arborization of the dendrites in repairing lesions, as opposed to the lower arborization found in demyelinating lesions (15, 16). We cannot completely rule out, however, that the increase in CME observed at follow-up could have been, at least in part, influenced by resolution of possible inflammatory processes within cortical lesions (28).

A lower information processing speed was associated with lower CME in several regions, especially in the right superior and inferior parietal, lateral occipital, and post-central cortex and in the left rostral middle frontal, superior frontal, superior parietal, and posterior cingulate. These areas are involved in spatial orientation, object recognition, working memory, and executive attention.

No voxel-wise correlations with EDSS were found, probably due to the low scores and scarce variability in the EDSS in our early cohort (mean EDSS ± SD was 1.8 ± 1).

Limitations to our study include the small follow-up sample that might have affected the statistical power to investigate further longitudinal changes in cortical CME, as well as the lack of longitudinal data for healthy subjects, although high scan-rescan reproducibility of CME in healthy subjects was previously demonstrated (14).

In conclusion, CME from ultra-high-resolution quantitative T${}_{2}^{*}$ and T_1_ at 7T is a sensitive technique for detecting, in early MS, cortical microstructural abnormalities related to myelin changes. This promising technique finds further application in other neurological diseases caused by altered cortical integrity.

## Data Availability Statement

The original contributions presented in the study are included in the article/supplementary material, further inquiries can be directed to the corresponding author/s.

## Ethics Statement

The studies involving human participants were reviewed and approved by Mass General Brigham Institutional Review Board. The patients/participants provided their written informed consent to participate in this study.

## Author Contributions

CM and VB contributed to conception and design of the study. VB performed statistical analysis and wrote the first draft of the manuscript. VB, EH, CT, AM, and RO contributed to data acquisition and analysis. All authors contributed to manuscript revision, read, and approved the submitted version.

## Conflict of Interest

The authors declare that the research was conducted in the absence of any commercial or financial relationships that could be construed as a potential conflict of interest.

## Publisher's Note

All claims expressed in this article are solely those of the authors and do not necessarily represent those of their affiliated organizations, or those of the publisher, the editors and the reviewers. Any product that may be evaluated in this article, or claim that may be made by its manufacturer, is not guaranteed or endorsed by the publisher.
